# Renal Lymphatics: Anatomy, Physiology, and Clinical Implications

**DOI:** 10.3389/fphys.2019.00251

**Published:** 2019-03-14

**Authors:** Peter Spencer Russell, Jiwon Hong, John Albert Windsor, Maxim Itkin, Anthony Ronald John Phillips

**Affiliations:** ^1^Applied Surgery and Metabolism Laboratory, School of Biological Sciences, University of Auckland, Auckland, New Zealand; ^2^Surgical and Translational Research Centre, Faculty of Medical and Health Sciences, University of Auckland, Auckland, New Zealand; ^3^Center for Lymphatic Disorders, Penn Medicine, Perelman School of Medicine, University of Pennsylvania, Philadelphia, PA, United States

**Keywords:** lymphatic system, kidney, anatomy, physiology, acute kidney injury, edema

## Abstract

Renal lymphatics are abundant in the cortex of the normal kidney but have been largely neglected in discussions around renal diseases. They originate in the substance of the renal lobule as blind-ended initial capillaries, and can either follow the main arteries and veins toward the hilum, or penetrate the capsule to join capsular lymphatics. There are no valves present in interlobular lymphatics, which allows lymph formed in the cortex to exit the kidney in either direction. There are very few lymphatics present in the medulla. Lymph is formed from interstitial fluid in the cortex, and is largely composed of capillary filtrate, but also contains fluid reabsorbed from the tubules. The two main factors that contribute to renal lymph formation are interstitial fluid volume and intra-renal venous pressure. Renal lymphatic dysfunction, defined as a failure of renal lymphatics to adequately drain interstitial fluid, can occur by several mechanisms. Renal lymphatic inflow may be overwhelmed in the setting of raised venous pressure (e.g., cardiac failure) or increased capillary permeability (e.g., systemic inflammatory response syndrome). Similarly, renal lymphatic outflow, at the level of the terminal thoracic duct, may be impaired by raised central venous pressures. Renal lymphatic dysfunction, from any cause, results in renal interstitial edema. Beyond a certain point of edema, intra-renal collecting lymphatics may collapse, further impairing lymphatic drainage. Additionally, in an edematous, tense kidney, lymphatic vessels exiting the kidney via the capsule may become blocked at the exit point. The reciprocal negative influences between renal lymphatic dysfunction and renal interstitial edema are expected to decrease renal function due to pressure changes within the encapsulated kidney, and this mechanism may be important in several common renal conditions.

## Introduction

Lymphatic vessels throughout the body begin as blind-ended capillaries in the interstitial space. They coalesce to form larger collecting vessels that eventually drain into veins. In this way, they serve a vital function throughout the body, including the kidney, in draining fluid and macromolecules from the interstitial space and returning them to the systemic circulation. This prevents accumulation of interstitial fluid that would impair oxygen delivery to the tissues. The kidney is richly supplied with lymphatic vessels, suggesting a key role of the renal lymphatic system in normal and stressed physiological states (Kaiserling, 1940). However, the role of the renal lymphatics in disease, and the anatomy and physiology of renal lymphatics in general, has been largely overlooked in recent decades.

Lymphatic dysfunction is defined as a failure to adequately drain interstitial fluid, and may have a variety of causes (e.g., valve failure, lymphatic vessel obstruction, loss of pressure gradients or loss of smooth muscle contractility). This manifests clinically as peripheral pitting edema when present in a limb, but is less obvious in the kidney. However, due to rigidity of the renal capsule, failure of lymphatic drainage may lead to raised intra-renal pressure and contribute to renal dysfunction. We suspect that this overlooked mechanism is present in a variety of clinical scenarios, such as congestive heart failure (CHF), AKI in the setting of the SIRS, chronic renal failure, renal transplant graft failure, and others.

This amounts to many clinical scenarios in which renal lymphatics may have an important role, yet there is a surprising scarcity of information on this important topic in the recent literature. Here we aim to comprehensively review the state of knowledge of renal lymphatics. We begin by reviewing the comparative anatomy in humans and animals. This is followed by a discussion of the physiology of the renal lymphatic system under normal and stressed conditions, then a summary of the implications of renal lymphatic dysfunction in various disease states.

## Anatomy of Renal Lymphatics

### Methods of Studying the Anatomy of Renal Lymphatics

It is thought that Paolo Mascagni made the first description of renal lymphatics in 1787 after injecting mercury into kidneys of cadavers (Mascagni, 1787). Since then, most studies have used dye (e.g., tryptan blue, India ink, Evans blue dye), injected intravenously or into the renal parenchyma, to examine renal tissue microscopically. However, in recent decades, the study of renal lymphatic anatomy has greatly advanced with improvements in imaging techniques and the development of LEC markers.

#### Lymphatic Endothelial Cell Markers

Although LEC markers are not specific to LECs, they can differentiate them from blood vessel endothelial cells. The most widely used markers are podoplanin, the LYVE-1, vascular endothelial growth factor receptor 3 (VEGFR-3), and the prospero-related homeo-box transcription factor 1 (Prox1) (Seeger et al., 2012). Of these, podoplanin, a mucin-type transmembrane protein, is the most reliable in human kidneys and can be localized in paraffin-embedded tissue using podoplanin antibody (Kalof and Cooper, 2009).

#### Imaging of the Renal Lymphatic System *in vivo*

Renal lymphatic anatomical studies, whether by microscopic or microradiographic technique, have previously required removal of the kidney. However, techniques for visualizing larger lymphatics *in vivo* have been developed and are currently in use (e.g., pedal lymphangiography, lymphangiography and lymphoscintigraphy) (Itkin and Nadolski, 2018), although current methods do not allow visualization of renal lymphatics. It is, however, possible to image the central lymphatic system by injection of contrast into inguinal lymph nodes (intranodal lymphangiography), and direct trans-abdominal catheterization of the cisterna chyli (Itkin and Nadolski, 2018). Minimally invasive lymphatic procedures have also been described. Injection of embolization material, such as N-butyl cyanoacrylate (N-BCA) glue (TRUFILL, Codman Neuro, Rayhnam, MA, United States), into the interstitium of lymph nodes can lead to propagation and embolization downstream (Itkin and Nadolski, 2018). Liver lymphatics can be imaged by contrast injection into the peri-portal space (Itkin et al., 2017). Taken together, this indicates renal interstitial injection of contrast (or probe) material for imaging renal lymphatics could be feasible in the future.

### Embryological Anatomy

Sabin (1909) first proposed that LECs arise by sprouting from embryonic veins, which has now been confirmed by lineage tracing studies using LEC markers (Tammela and Alitalo, 2010). Using expression of LYVE-1, Lee et al. (2011) studied the development of renal lymphatics in mice embryos. Intra-renal lymphatics, connected to extra-renal lymphatic plexuses, were first detected in the 13-day old embryo. These lymphatics formed well-organized networks that followed the developing arcuate and interlobular vessels over the next few days. None were observed in the renal medulla at any developmental stage.

Lee et al. (2011) noted that scattered LYVE-1^+^ cells were observed in the developing arcuate veins, mainly in the branching buds, and that lymphatic vessels were mainly located around developing veins until post-natal day 4. Immature macrophages and dendritic cells also expressed LYVE-1 and appeared prior to LYVE-1^+^ lymphatic vessels. These cells were seen closely intermingled or even forming part of the lymphatic vascular wall. They concluded that LYVE-1^+^ cells initially bud off from veins but that LYVE-1^+^ macrophages and dendritic cells are crucial in orchestrating a branching process that connects these cells to extra-renal lymphatic vessels. VEGF-C also seems to have an essential regulatory role in this branching process (Lee et al., 2011; Mohamed and Sequeira-Lopez, 2018). Tanabe et al. (2012) similarly found that renal lymphatic vessels in the rat develop initially from pre-existing extrarenal lymphatic vessels and extend toward the cortex along the renal blood vasculature. Intra-renal lymphatic vessels were first seen at embryonic day 20. It is still unclear whether the mammalian kidney possesses intrinsic lymphatic precursors that contribute to the development of intra-renal lymphatic vessels (Mohamed and Sequeira-Lopez, 2018).

### Renal Vascular Anatomy

**FIGURE 1:**
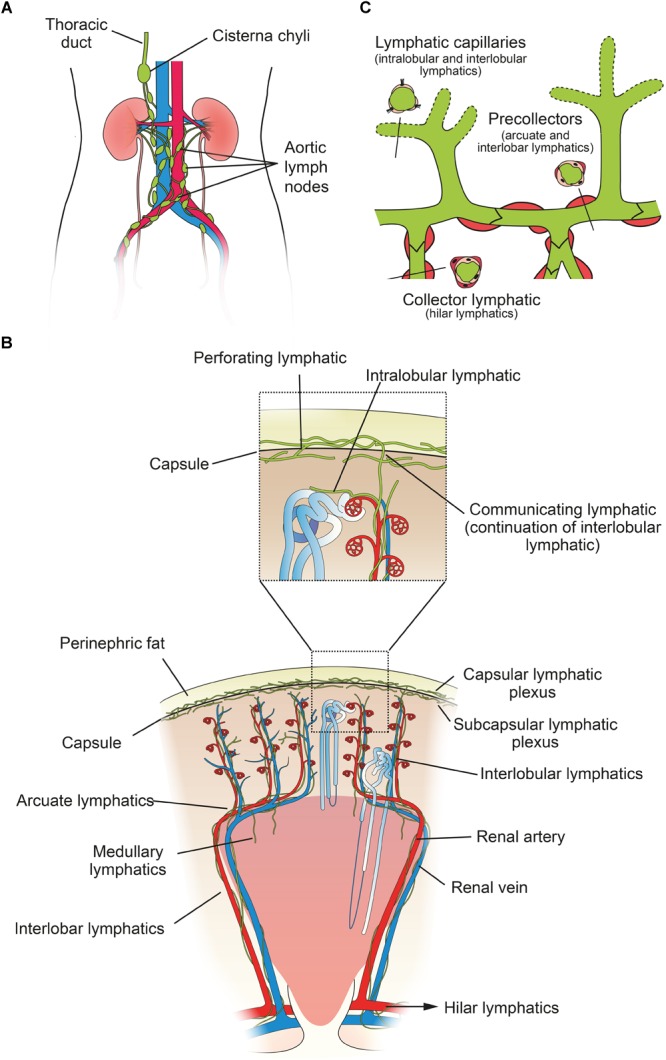
Structure of the human renal lymphatic system. **(A)** Lymph passes from 4–5 renal hilar lymphatics on each kidney to various groups of aortic lymph nodes. Most lymph draining from the kidney collects in the cisterna chyli and is drained via the thoracic duct into the central venous circulation in the neck. **(B)** Schematic diagram of a human renal lobe. Blind-ended lymphatic capillaries begin in the substance of a renal lobule as intralobular lymphatics, which in turn become interlobular lymphatics. From here, lymph can flow either toward the capsule or toward the hilum. Valves are present at the level of the capsule and arcuate vessels to prevent backflow. Intra-renal arteries in red and intra-renal veins in blue. Insert shows detail of communicating and perforating lymphatics that pierce the renal capsule. **(C)** Schematic showing morphology of renal lymph vessels. Lymphatic vessels vary morphologically between initial capillaries (no basement membrane or smooth muscle), pre-collectors (some smooth muscle and occasional valves) and collectors (continuous basement membrane and smooth muscle layer, valves present). Figure based on information from O’Morchoe and O’Morchoe (1988) and Seeger et al. (2012).

Renal lymphatics follow the topography of the renal vasculature (O’Morchoe and O’Morchoe, 1988) and therefore a brief review of the arterial supply to the kidney is pertinent. There is usually a single renal artery supplying each kidney. In the renal sinus it divides into five segmental arteries (posterior, superior, anterosuperior, anteroinferior, and inferior) (Bhimji and Leslie, 2018). Each segment of the kidney is made up of *lobes* (consisting of a renal pyramid overlaid by a portion of renal cortex), which is then divided into renal *lobules* (consisting of the nephrons surrounding a single medullary ray and draining into a single collecting duct). The arteries arising from a segmental artery follow this lobular pattern (Figure 1). An interlobar artery radiates out between lobes, and gives rise to arcuate arteries that traverse the cortico-medullary junction. From each arcuate artery arises an interlobular artery that radiates between lobules toward the capsule. These give off afferent arterioles leading toward the glomerulus. The venous system is the same as the arterial, with the exception that there is communication between veins from different segments, whereas the segmental arteries are end arteries.

### Comparative Renal Lymphatic Anatomy

#### Renal Lymphatics in Non-mammalian Vertebrates

The lymphatic system probably evolved around the time aquatic organisms moved onto land, as there is no well-developed lymphatic system in fish but there is in amphibians (Brown, 2005). Reptiles, many amphibians and bird embryos have lymph ‘hearts’ to assist with moving lymph. These are pulsating chambers, located at points where lymph vessels enter veins. They are highly variable in wall thickness, contain a valve and beat independently of the blood heart rhythm (Satoh and Nitatori, 1980). The kidneys of fish and anuran amphibians have an accessory role as a lymphoid organ, whereas this becomes less so in the kidneys of amniotes (Manning, 1979). In general, reports of reptile (e.g., Davis et al., 1976) and avian (e.g., Morild et al., 1985) renal anatomy fail to mention renal lymphatics.

#### Mammalian Renal Lymphatic Anatomy

The majority of research on renal lymphatic anatomy has been done on mammals, particularly dogs. In a seminal study, Pierce (1944) injected dog, rabbit and guinea pig kidneys with India ink, as well as giving intravenous tryptan blue, in order to delineate the renal lymphatics microscopically. He found that renal lymphatics begin in the cortex as *intralobular lymphatics*, which are sparse, blind-ended tubes in close proximity to the renal tubules. These pass close to, but do not enter, a renal corpuscle, before joining the *interlobular lymphatics*, which connect to the larger *arcuate* and *interlobar lymphatics*. These then drain into hilar lymphatics (Figure 1). There are 6–8 hilar lymphatics and 4–6 lymphatic channels that leave the renal capsule in the dog (Cockett, 1977). Capsular lymphatics, after leaving the capsule, appear to connect to hilar lymphatics in the renal sinus and hilum (Yazdani et al., 2014). The total volume of cortical lymph in a dog kidney is approximately equivalent to only 1% of the volume of blood in the cortical peritubular capillaries (O’Morchoe and Albertine, 1980).

Anatomy between different species is very similar with a few exceptions; rabbit kidneys appear to lack intralobular lymphatics (O’Morchoe and O’Morchoe, 1988) and the sheep kidney lacks a capsular system (McIntosh and Morris, 1971) (Table 1). The species with the least extensive intra-cortical lymphatics (the rabbit) has the lowest urine concentrating ability and the species with the most extensive system (the golden hamster) has the highest concentrating ability (Niiro et al., 1986).

**Table 1:** Comparison of renal lymphatic anatomy between species.

Species	Intralobular lymphatics	Medullary lymphatics	Communication between renal and capsular lymphatics	Glomerular lymphatics	Comments	Key references
Dog	Present	*Present* (Cockett, 1977) or *Absent* (Pierce, 1944; Bell et al., 1968; Albertine and O’Morchoe, 1980; Eliska, 1984)	Present	Partially surround Bowman’s capsule	Species most extensively studied	Pierce, 1944; Bell et al., 1968; Holmes et al., 1977a; O’Morchoe and Albertine, 1980
Pig	Present	Present	Present			Clark and Cuttino, 1977; Cuttino et al., 1985
Rat	Intermediate	Not found		Lymphatics lie close to glomerulus	Intrarenal lymphatic vessels appear at embryonic day 20	Niiro et al., 1986; Tanabe et al., 2012
Mouse			Present		Intrarenal lymphatic vessels appear at embryonic day 13	Holmes et al., 1977a; Lee et al., 2011
Rabbit	Rare	Present	Not found		Least extensive intralobular lymphatics and lowest urine concentrating ability	Kaiserling, 1940; Pierce, 1944; Ruszniják et al., 1960; Niiro et al., 1986
Guinea Pig	Extensive				Most extensive intralobular lymphatics and highest urine concentrating ability	Pierce, 1944; Niiro et al., 1986
Horse				Completely surround Bowman’s capsule		Bell et al., 1968
Sheep		Not found	Absent (no capsular lymphatics)			McIntosh and Morris, 1971
Human	Present	Rare and in outer medulla only or surrounding vasa recta	Present	Sporadically surround glomerulus	Medullary lymphatics seen in pathological specimen	Rawson, 1949; Cuttino et al., 1989; Ishikawa et al., 2006

*Space left blank if no data were found.*

#### Human Renal Lymphatic Anatomy

**FIGURE 2:**
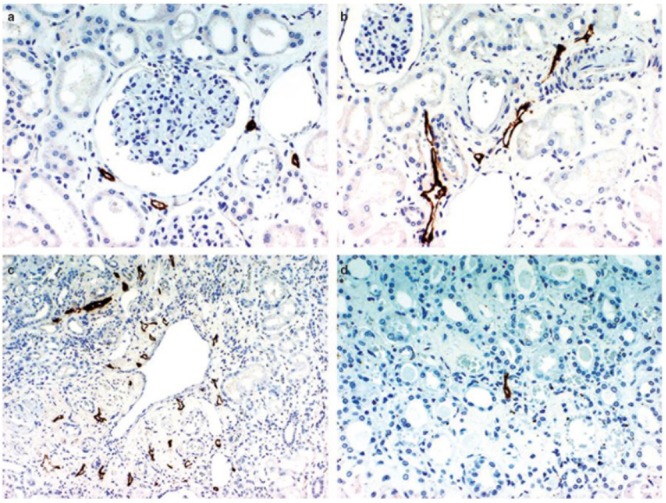
D2-40 immunostaining of lymphatics in the normal kidney. **(a)** Lymphatic capillaries in the interstitium around the glomerulus, **(b)** lymphatics exhibiting a slit-like structure are distributed around the interlobular artery and vein in the cortex, **(c)** multiple lymphatic capillaries in the interstitium around a dilated interlobular vein, a few lymphatic capillaries are present just beneath the venous endothelium, **(d)** a lymphatic capillary is recognizable in the center of the figure showing a normal medulla. Reproduced with permission from Ishikawa et al. (2006).

The basic mammalian renal lymphatic anatomy outlined above is the same as that found in humans (Figure 1). This was shown with microscopy at autopsy (Rawson, 1949) or using radiographic studies (Cuttino et al., 1989), and has been confirmed using LEC markers. Ishikawa et al. (2006), using podoplanin antibody (Figure 2), studied normal human kidney tissue obtained at autopsy, and confirmed that lymphatic vessels were most abundant in the interstitium surrounding interlobular, arcuate and interlobar arteries and veins.

From the left kidney lymphatic vessels enter preaortic, para-aortic and retroaortic lymph nodes, and from the right kidney, they enter paracaval, precaval, retrocaval and interaortocaval lymph nodes (Karmali et al., 2014). Both kidneys also send lymphatics posterior to the aorta, which can travel directly to the thoracic duct (Assouad et al., 2006). The surface of the upper pole may also drain through the diaphragm into posterior mediastinal lymph nodes. Renal lymphatics may reach very distant nodes, but always eventually connect to the origin of the thoracic duct, usually via the cisterna chyli (Assouad et al., 2006) (Figure 1).

The terminal thoracic duct variably drains into the left internal or external jugular vein, the left subclavian vein or the venous angle (Ratnayake et al., 2018). The ostial valve is found at this junction and prevents backflow of venous blood (Ratnayake et al., 2018).

### Detailed Features of Human and Mammalian Renal Lymphatic Anatomy

#### Communication Between Intra-Renal and Capsular Lymphatics

Pierce (1944) found a connection between cortical and capsular/perirenal lymphatics in dogs suggesting that lymph can leave the kidney through an alternative route, rather than the renal hilum. Evans blue dye injected into the renal parenchyma appears in hilar, or capsular lymphatics, or both (Sugarman et al., 1942). This lymphatic communication has been confirmed in other species (Bell et al., 1968; O’Morchoe, 1985), including humans (Rawson, 1949), and recently using LEC markers (Seeger et al., 2012), but Kaiserling (1940) was unable to detect this communication in rabbits.

Holmes et al. (1977a) described two sets of connecting lymphatics in dogs, which are differentiated based on where they penetrate the capsule (Figure 1). The first are *perforating lymphatics*, which are the majority. They penetrate the capsule at any point, either alone or with a small vein, from the subcapsular plexus. They form the primary pathway for lymph from the most superficial parts of the cortex, as opposed to the hilar route (the hilar route is the primary path for the majority of renal lymph). The second are *communicating lymphatics*, which form the main connecting link between the hilar and capsular systems. These are a continuation of interlobular lymphatics and are closely associated with occasional interlobular arteries and veins that penetrate the capsule. Each kidney was found to contain only 5–10 interlobular arteries that penetrated the capsule like this, and of these, 60% were accompanied by a communicating lymphatic. Where it pierced the capsule the lymphatic was single, but either side of the capsule it quickly branched to form either the interlobular lymphatic plexus or the capsular plexus. For excellent histological sections of communicating and perforating lymphatic vessels in a dog, see Holmes et al. (1977a).

#### Medullary Lymphatics

Pierce (1944) found no evidence of medullary lymphatics in dogs, consistent with the lack of arterial supply in the medulla, and concluded they do not exist. Since Pierce, most animal studies have failed to detect medullary lymphatics. McIntosh and Morris (1971), using light and electron microscopy, as well as physiological studies, found no evidence of medullary lymphatics in the sheep. Bell et al. (1968), Albertine and O’Morchoe (1980), and Eliska (1984) could not detect medullary lymphatics in dogs. In contrast to these findings, Cuttino et al. (1985) reported a medullary lymphatic system in pigs, Cockett (1977) in dogs and Ruszniják et al. (1960) in rabbits. Animal studies examining content of renal lymph have shown that medullary interstitial fluid must contribute to hilar lymph (Keyl et al., 1972), suggesting the presence of medullary lymphatics. However, fluid movement within the medullary interstitium to juxtamedullary lymphatics (in the cortex) can also cause this result (Albertine and O’Morchoe, 1980; O’Morchoe and O’Morchoe, 1988).

Rawson (1949) mapped lymphatic distribution in a human kidney autopsy specimen. The specimen was from a patient with a gastric carcinoma that had metastasized in a retrograde manner into the renal lymphatics. This dilated the lymphatics and made them visible under the microscope. He concluded that renal lymphatics begin blindly in two areas; near Bowman’s capsule in the cortex, and beneath the mucosa of the papilla in the medulla. Those in the cortex drain down toward the arcuate vessels and those in the medulla drain up to meet them. Rawson’s findings have been controversial due to the presence of cancer in the renal lymphatics, and recent review articles have not relied on his work and instead have suggested, based on animal studies, that no lymphatic vessels are present in normal human renal medulla (Lee et al., 2011; Seeger et al., 2012). However, Ishikawa et al. (2006) found medullary lymphatics in normal human renal tissue in four out of ten cases (Figure 2), but only near the cortex, with none seen in the central area of the medulla.

#### Glomerular and Tubular Lymphatics

Bell et al. (1968) found numerous small lymphatic vessels intimately associated with glomeruli, but none that penetrated into the glomerulus. These vessels either completely (horse) or partially (dog) surrounded Bowman’s capsule. Eliska (1984) was able to detect rare and very small lymphatics near tubules between interlobular arteries, and noted that lymphatics surrounding glomeruli become rarer with increasing distance from an interlobular artery. Ishikawa et al. (2006) confirmed that lymphatic capillaries sporadically surround the glomerulus in humans but do not penetrate it (Figure 2).

#### Valves

Bi-leaflet valves are seen in pre-collector (arcuate and interlobar lymphatics) and collecting (hilar) lymphatics, and facilitate unidirectional flow toward the hilum and aortic lymph nodes (Seeger et al., 2012) (Figure 1). For communicating and perforating lymphatics, often a single valve is present as they traverse the capsule, oriented to prevent flow from the surface back into the renal parenchyma (Holmes et al., 1977a). These valves continue into capsular lymphatics and are numerous there. No valves are present in interlobular lymphatics (Holmes et al., 1977a). Thus, lymph forming in the cortex is permitted to exit the kidney in either direction.

#### Renal Interstitium

The renal interstitium is defined as the intertubular, extraglomerular, extravascular space of the kidney (Zeisberg and Kalluri, 2015). It is bound on all sides by tubular and vascular basement membranes. The cortical renal interstitium is divided into (a) peritubular interstitium, (b) periarterial interstitium, and (c) glomerular and extra-glomerular mesangium (Lemley and Kriz, 1991). The lymphatics lie in the periarterial interstitium, which is a fluid-rich loose connective tissue sheath. It is seen surrounding afferent arterioles and larger arteries, but it is less distinct in efferent arterioles and tubular capillaries.

#### Morphology of Renal Lymph Vessels

Renal lymphatic capillaries are considered part of the interstitium because they lack a basement membrane. They are differentiated from blood vessels in that they are blind-ended and lack pericytes. LECs of capillaries are single-layered, ‘oak-leaf’-shaped, partly overlapping cells, interconnected via discontinuously arranged ‘button-like’ junctions, which result in interjunctional gaps (Seeger et al., 2012). These junctions are composed of vascular endothelial cadherin (VE-cadherin) and various tight junction-associated proteins (e.g., occludin, zonula occludens-1) (Baluk et al., 2007). LECs are connected to the perivascular matrix by filaments. This arrangement leads to an increase in diameter of the vessel lumen and width of intercellular gaps in the presence of tissue edema, which facilitates fluid entry (Seeger et al., 2012). Precollectors (arcuate and interlobar lymphatics) exhibit some perivascular smooth muscle cells, and collectors (hilar lymphatics) contain continuous endothelial junctions, a basement membrane and a smooth muscle cell layer (Figure 1). Lymphatic vessels usually transport lymph via both extrinsic pumping, secondary to surrounding tissue forces, and intrinsic pumping, via these smooth muscle cells, with the latter likely predominating in renal lymphatics (Zawieja, 2009).

#### Relationship to Arteries and Veins

Ishikawa et al. (2006) showed that, in humans, lymphatics around interlobular veins were more developed than lymphatics around interlobular arteries. They saw abundant lymphatics in the interstitium around interlobar arteries but noted that lymphatics surrounding interlobar veins were distributed not just in the interstitium, but also in the wall itself, in both the media and the intima just under the endothelium. Although lymphatics travel close to renal vessels, Eliska (1984) and others have been unable to detect any anatomical intra-renal lymphatic-venous shunts. Lymphovenous communications have been seen, however, at the level of the renal vein in rats and primates, but not in human autopsy studies (Karmali et al., 2014).

### Summary of Human Lymphatic Anatomy of the Kidney

Human renal lymphatic anatomy has the same overall structure as other mammals. Blind-ended lymphatic capillaries begin near renal tubules, pass close to the glomerulus, and then follow the renal arteries as interlobular, arcuate and interlobar lymphatics. In the cortex, interlobular lymphatics do not possess valves, and so lymph is able to travel along the primary route toward the hilum, or toward the capsular lymphatic plexus, penetrating the capsule. Therefore, lymph is able to exit the kidney via two different routes. Lymphatic capillaries are relatively abundant in the cortex but very rare in the medulla. They are tethered to the surrounding interstitium, lack a basement membrane and have interjunctional gaps between endothelial cells. Larger arcuate, interlobar and hilar lymphatics possess valves for unidirectional flow and smooth muscle to aid pumping of lymph.

## Renal Lymphatic Physiology Under Normal Conditions

### Methods of Studying the Physiology of Renal Lymphatics

Similar to the published studies of renal lymphatic anatomy, physiology studies have relied heavily on animals. Experimental *in vivo* sampling of renal lymph has been achieved in dogs (LeBrie and Mayerson, 1960) and rats (Hargens et al., 1977), but not in humans. Measuring renal lymphatic flow rates in animals has proven difficult due to the anatomical arrangement of lymphatics in the kidney, but has been achieved and will be discussed in the relevant sections below.

### Formation of Renal Lymph

**FIGURE 3:**
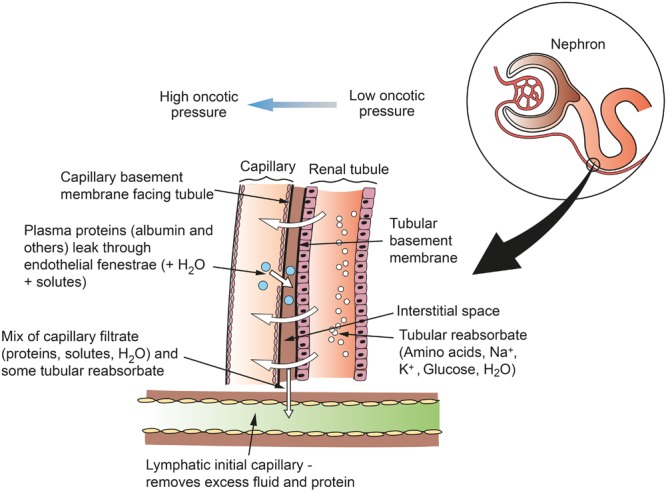
Schematic of flows of reabsorbate from renal tubule to capillary and of protein from capillary to lymph in the renal cortex. Fenestrations on the capillary wall are clustered near narrow interstitial spaces between the capillary and the tubule. Lymph is formed in the interstitial space and taken up into lymphatic capillaries. This mechanism does not apply in the medulla, where lymphatics are sparse and interstitial fluid and macromolecules are taken up into the vasa recta. Figure based on information from Pinter (1988).

Lymphatic vessels remove fluid and macromolecules from the interstitial space between tubules and capillaries. Lymph enters lymphatic capillaries according to the balance between hydrostatic and oncotic pressures according to the Starling equation (Staub and Taylor, 1984). All blood capillaries leak proteins into the interstitium, and especially so if endothelial fenestrations are present. In the kidney, fenestrations are more common on the side of the capillary wall that faces the renal tubule, as the capillary and tubule lie close together (Pinter, 1988). Therefore, as re-uptake of proteins into the capillary is not complete, plasma proteins are likely to accumulate in the narrow interstitial space between the capillary and the tubule (Figure 3). If proteins entering the interstitium are not removed, the important oncotic pressure gradient would disappear, leading to a decrease in the flow of fluids and electrolytes from tubules to peri-tubular capillaries (Cockett, 1977; Pinter, 1988). The kidney is unique among organs in having a large extravascular albumin pool that is rapidly removed and replenished. Slotkoff and Lilienfield (1967) demonstrated this by intravenously infusing radioactively labeled red cells and albumin into dogs. They then perfused the kidneys with dextran (MW 75,000) and measured the remaining tissue radioactivity. They found a greater proportion of labeled albumin remained in the kidneys compared with labeled red cells, and concluded that a significant extravascular albumin pool exists. This difference was greatest in the medulla where there are fewer lymphatics to remove the albumin. Interstitial albumin in the medulla is necessary for the kidney’s urine concentrating ability. In the cortex, removal of interstitial albumin is a primary function of the lymphatics, where, as mentioned above, the oncotic pressure gradients are necessary for tubular reabsorption.

Interstitial fluid and proteins flow freely into terminal lymphatics down hydrostatic and oncotic pressure gradients. Renal interstitial fluid pressure is positive, unlike that of subcutaneous tissue or muscle, which results in a relatively high normal lymph flow (Ott and Knox, 1976). Entry into lymphatics is mainly via interjunctional gaps between LECs, but these endothelial cells are highly endocytic and are also engaged in transcellular uptake of fluid and macromolecules (Seeger et al., 2012). Entry is also aided by tethering filaments and a lack of a basement membrane. Filaments connect LECs to the surrounding perivascular matrix and therefore increase the size of interendothelial gaps when the tissue is edematous. The composition of lymph in the terminal lymphatic is almost the same as interstitial fluid from which it is derived.

#### Formation and Flow of Capsular Lymph Versus Hilar Lymph

Lymph from the most outer part of the cortex is primarily drained via the capsular lymph vessels, in contrast to lymph from the medulla and remainder of the cortex, which is primarily drained via hilar lymphatics (O’Morchoe et al., 1975). Although most renal lymph is formed in the cortex, flow rate from hilar lymph has been measured at 4–8 times greater than capsular lymph (Keyl et al., 1965), suggesting that most cortical lymph drains via hilar, and not capsular, lymphatics. This is further evidenced by the fact that the electrolyte composition of hilar lymph is very similar to plasma, which is because the electrolyte composition of the cortical interstitium (where most hilar lymph is formed) also closely resembles plasma. These solutes are found in high concentrations in medullary interstitium (due to the requirements of the counter-current mechanism for concentrating urine), and renal hilar lymph would reflect these concentrations if it were primarily draining the medulla (Keyl et al., 1965).

### Composition of Renal Lymph Under Normal Conditions

#### Renal Lymph Derives From Capillary Filtrate and Tubule Reabsorbate

Studies have confirmed that renal lymph is derived from both capillary filtrate and tubule reabsorbate (Bell and Lowsitisukdi, 1983; Bell, 1984). Kaplan et al. (1942) gave intravenous inulin to dogs and found a renal inulin lymph/arterial plasma ratio of 0.68. If renal lymph where derived exclusively from renal tubular reabsorbate, lymphatic inulin content would be close to zero as inulin is not reabsorbed. Other studies have found a lymph/plasma ratio of 0.80 for inulin (Keyl et al., 1965), 0.78 for creatinine (Cockett et al., 1969) and 0.58 for para-aminohippurate (PAH) (Keyl et al., 1965). The mixed source of renal lymph can also be shown by measuring the ratio of labeled glucose (reabsorbed by the proximal tubule) and mannitol (not reabsorbed) in renal lymph compared with plasma (Cook et al., 1982). The labeled glucose appears in renal lymph in a higher proportion compared with mannitol after reabsorption from the proximal tubule, indicating that tubule reabsorbate contributes to the formation of renal lymph.

If renal lymph were derived solely from capillary filtrate, then its electrolyte composition would be identical to plasma (Cockett, 1977), and while renal lymph biochemistry does resemble plasma, it is not identical. LeBrie and Mayerson (1959) found capsular lymph had an 11.3 and 26.9% higher concentration of Na^+^ and Cl^-^, respectively, compared with plasma. They concluded that the distal tubule is able to reabsorb solute independently of water, and this concentrated solution of Na^+^ and Cl^-^ is mixed with capillary filtrate to make renal lymph. This was not confirmed by Mayerson (1963) who suggested that the sodium was erroneously high and not significant. Other studies have found similar concentrations of Na^+^, K^+^, Mg^2+^, osmolality and pH in plasma and renal lymph (Swann et al., 1958; LeBrie and Mayerson, 1959; Keyl et al., 1965). The concentration of urea in renal lymph varies between studies, with lymph/plasma ratios of 1.4 (Swann et al., 1958), 1.3 (Sugarman et al., 1942) and 0.89 (Keyl et al., 1965), and this discrepancy may represent a difference in methodologies. Keyl et al. (1965) speculated that a raised urea lymph/plasma ratio is due to clamping of capsular lymphatics in order to engorge them for cannulation. This impairs normal renal lymph drainage, which leads to variations in interstitial urea. These studies all suggest that capillary filtrate rather than tubule reabsorbate, is the major source of renal lymph.

#### Renal Lymph Proteins

Protein concentration in renal lymph varies greatly and has been measured at 20% (rat) (Hargens et al., 1977), 34% (dog) (Keyl et al., 1965), 49% (dog) (Atkins et al., 1988), 50% (dog) (LeBrie and Mayerson, 1960), 60% (dog) (LeBrie and Mayerson, 1959), and 66% (dog) (Henry et al., 1969) of systemic plasma levels, and 70% of thoracic duct lymph levels (LeBrie and Mayerson, 1959). Protein content of renal lymph varies with flow rate (Sugarman et al., 1942; LeBrie and Mayerson, 1960; Bell, 1985), with a lower protein concentration associated with an increased lymph flow rate. It also depends on whether the animal is anesthetized or not, with a higher protein concentration with anesthesia, although the mechanism for this is unknown (Henry et al., 1969).

LeBrie and Mayerson (1959, 1960) found no difference in relative proportions of different proteins (albumin, alpha 1, alpha 2, beta, and gamma globulins) between renal lymph and plasma, but others found a greater proportion of albumin and alpha globulins in lymph (Swann et al., 1958; Keyl et al., 1965; Hargens et al., 1977). The renal lymphatic clearance of albumin has been calculated to be quite high (0.16 cm^3^/min/100 g kidney) which is consistent with the relatively high permeability of renal capillaries (Atkins et al., 1988) and the high levels of interstitial albumin (Slotkoff and Lilienfield, 1967).

Renal lymph contains a relatively high concentration of renin, which is not found in lymph from other organs or in renal vein blood (Lever and Peart, 1962; Moffat, 1981). This may be explained by lymphatics lying in close proximity to the juxtaglomerular apparatus, where renin is synthesized and acts in a paracrine fashion (Moffat, 1981). Likewise, levels of angiotensin II were found to be higher in renal lymph than in renal arterial or venous samples in dogs (Bailie et al., 1971). It was noted that partial occlusion of the renal artery (renal artery stenosis) causes an increase in the concentration of renin in renal lymph (Lever and Peart, 1962). Apolipoproteins (specifically ARP and A-IV), which presumably have a lipid transport role in the renal interstitium, are also present in renal lymph in relatively high concentrations (Roheim et al., 1976).

### Renal Lymph Flow Rates Under Normal Conditions

Total renal lymph flow rates are difficult to measure because renal lymphatics do not coalesce before entering the periaortic chain (Atkins et al., 1988). The variation in experimental measurements of renal lymph flow reflects this difficulty. It is thought that under normal conditions, lymph flow represents a small fraction of fluid leaving the kidney (Goodwin and Kaufman, 1956), but some reports suggest lymph flow equals urine flow under normal conditions (Stolarczyk and Carone, 1975). Sugarman et al. (1942) calculated lymph flow to be about 2% of fluid reabsorption in the kidney (which would roughly equate to urine flow). Cockett (1977) estimates lymph flow to be approximately one-half the volume of urine flow in dogs under baseline conditions. Atkins et al. (1988) calculated an average total renal lymph flow of 0.36 ml/min/100 g kidney, with each kidney contributing 21% of the thoracic duct flow in fasted dogs. The authors attributed this high value to the fact the animals were fasted and would be expected to have low cisterna chyla flows (Atkins et al., 1988).

### Interstitial Fluid and Protein Drainage in the Medulla

In contrast to the cortex, recycling of interstitial fluid and proteins from the medulla is not via lymphatics. Rather than diffusion to cortico-medullary lymphatics, they are mostly removed by the vasa recta. Tenstad et al. (2001) showed this by infusing labeled albumin into the inner medulla of rat kidneys and found it first appeared in plasma, rather than lymph. In contrast, albumin infused into the cortex was seen first in thoracic duct lymph. Transport into the vasa recta is likely from convective flow, where proteins are moved alongside fluid. Medullary fluid moves down its concentration gradient from the collecting ducts to the interstitium and then the vasa recta. This process can be explained by mathematical models (Wang and Michel, 2000). Tenstad et al. (2001) conclude that, like the brain, cornea and bone marrow, there is ‘simply no need for lymphatics in the inner medulla.’

## Renal Lymphatic Physiology in Pathological Conditions

### Composition of Renal Lymph in Pathological Conditions

#### Electrolytes

LeBrie and Mayerson (1960) studied the composition of renal lymph after partial occlusion of the inferior vena cava (mimicking right heart failure). They found no change in the concentration of Na^+^ and Cl^-^, even though overall lymphatic flow (and therefore total Na^+^ and Cl^-^ content) increased. Profuse diuresis causes decreased concentrations of Na^+^ and Cl^-^ in renal lymph, a change which is unaffected by initial plasma electrolyte concentrations (O’Morchoe et al., 1970; O’Morchoe et al., 1978).

#### Proteins

Increased venous pressure (from 22 to 30–35 cmH_2_O in dogs) causes a markedly disproportionate rise in renal lymphatic protein content (LeBrie and Mayerson, 1960). This is thought to be due to increased capillary protein loss when venous pressure is raised to this level (LeBrie and Mayerson, 1960). Protein content of renal lymph decreases during diuresis, which is a direct result of increased lymphatic flow rates (LeBrie, 1968).

### Role of Lymphatics in the Fluid Economy of the Kidney

#### ‘Safety Valve’ Function of Renal Lymphatics

The three routes that fluid can leave the kidney are venous, ureteral and lymphatic. These three systems bear relationships to each other and operate reciprocally (Katz and Cockett, 1959). Both obstruction of the upper ureter and the renal vein results in increased renal lymph pressure and flow, and ligation of lymphatic vessels results in increased urine flow (Cockett, 1977). The lymphatic system has been said to function as a ‘safety valve’ mechanism protecting the kidney from high intra-renal pressures, such as in raised IRVP or hydronephrosis (Goodwin and Kaufman, 1956).

#### Renal Lymph Flow During Diuresis

Schmidt and Hayman (1929) found that the discrepancy between renal arterial and venous flow during profuse diuresis in dogs was not fully accounted for by urinary production, and concluded the remainder was leaving the kidney via lymphatics. They showed that the kidney, during diuresis, was capable of producing a substantial amount of lymph. O’Morchoe et al. (1970) found increased lymph flow with mannitol diuresis but not mersalyl or furosemide. They concluded the effect of mannitol on lymph flow was due to the general increase in interstitial fluid rather than through any specific intra-renal consequence of the diuresis itself.

#### Renal Lymph Flow During Increased Venous Pressure

Increasing systemic venous pressure causes a decrease in urinary flow and urinary sodium concentration, however, this is likely due to changes in renal blood flow, resulting in a lower glomerular filtration rate (GFR), rather than a direct consequence of the venous pressure (LeBrie and Mayerson, 1960).

**FIGURE 4:**
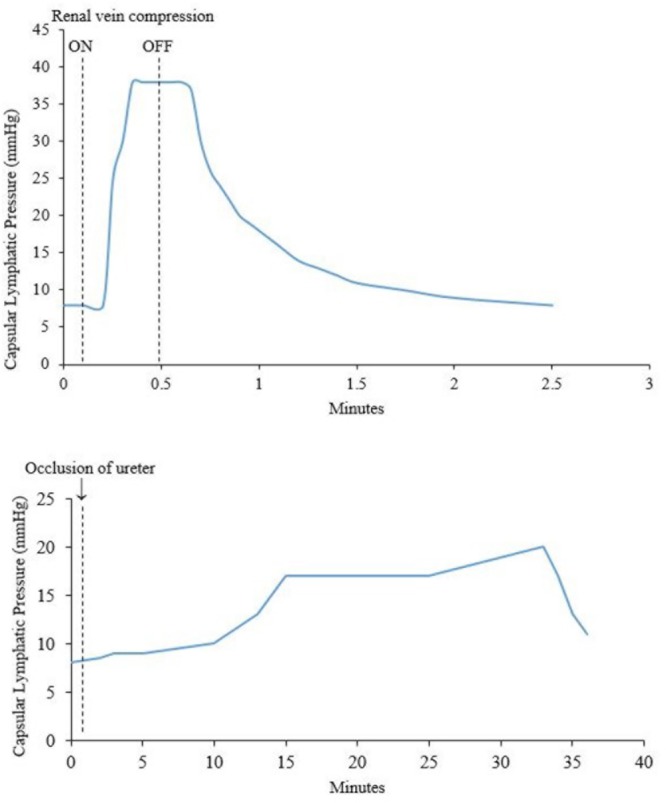
**(Top)** Effect of compression of the renal vein on renal capsular lymphatic pressure in a dog. Note that the rise in pressure occurs 5 s after compression of the renal vein. **(Bottom)** Effect of occlusion of the ureter on capsular lymphatic pressure in a dog. The ureter remained clamped throughout the time period of the graph. The sudden drop in pressure around 33 min was seen when the kidney was very tense and is presumably from occlusion of the lymphatic vessels at the point where they exit the kidney. Adapted with permission from Katz (1958).

Increased systemic venous pressure causes an increase in renal lymph flow. Katz (1958) measured the pressure of capsular lymphatics in dogs during occlusion of the renal vein or ureter. The lymphatic pressure began to rise sharply 5 s after renal vein occlusion, and declined 7 s after its release (Figure 4). A rise in interstitial pressure preceded the rise in lymphatic pressure. These changes also occurred in *partial* renal vein occlusion, a situation resembling the raised venous pressure seen in cardiac failure. LeBrie and Mayerson (1960) estimated that renal lymph flow increased up to 2400 ml/24 h from both kidneys under conditions of partial inferior vena cava (IVC) occlusion. This represents a volume 3–4 times greater than the plasma volume in a 15 kg dog.

**FIGURE 5:**
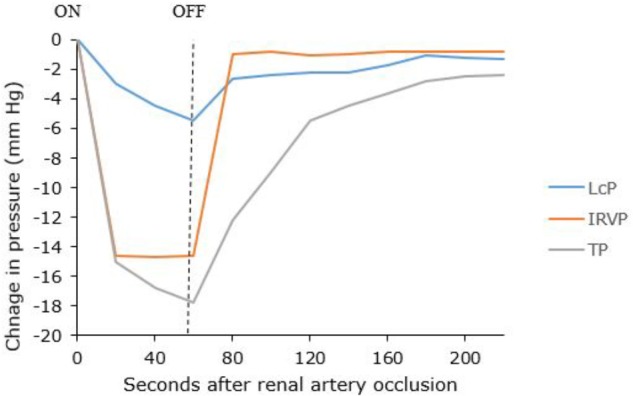
Changes in capsular lymph pressure (LcP), intra-renal venous pressure (IRVP), and tissue pressure (TP) before, during and after a 60-s renal artery occlusion. Adapted with permission from Bell et al. (1972).

IRVP and interstitial fluid volume are the main drivers of renal lymph formation (Bell et al., 1972, 1974; Keyl et al., 1972). Clamping the renal artery causes decreased capsular lymph pressure that reflects decreased IRVP and tissue pressure (Figure 5) (Bell et al., 1972). The IRVP quickly recovers when the artery is unclamped; however, the tissue pressure and lymphatic pressure recover more slowly. Bell et al. (1972) reasoned that tissue pressure was a result of an interstitial fluid volume that had to be replaced after the artery was unclamped. These experiments are relevant for patients with severe acute disease who have poor renal perfusion (e.g., circulatory shock). However, renal lymph and tissue pressures are maintained even in decreased renal blood flow as long as venous pressure is not allowed to fall, showing that IRVP is the major factor in renal lymph production (Bell et al., 1974).

#### Renal Lymph Flow During Hydronephrosis

Hydronephrosis also causes increased renal lymphatic flow (Goodwin and Kaufman, 1956). However, the rise in lymphatic pressure is more gradual after occlusion of the ureter, compared with occlusion of the renal vein (Katz, 1958) (Figure 4). Bell et al. (1974) noted that renal pelvic pressure is relatively ineffective at increasing lymphatic pressure and flow under conditions of decreased renal blood flow. Therefore, the increase in lymphatic pressure and flow seen in increased pelvic pressure is probably actually due to intra-renal venous obstruction secondary to the increased pelvic pressure. Increased pelvic pressure has been observed to obstruct venous outflow from the kidney due to the intimate intra-renal anatomical relationship between renal veins and pelvis (Keyl et al., 1972; Holmes et al., 1977b). In dogs, ureteric obstruction causes a diversion of hilar lymph to capsular lymph within 3 days due to compression of hilar lymphatics in the renal sinus by the distended renal pelvis (Holmes et al., 1977b).

Figure 4 shows a sudden decrease in capsular lymphatic pressure when the kidney was visibly very tense. The author concluded this was from occlusion of the capsular lymphatic vessels as they exited the kidney. This demonstrates a way that capsular renal lymphatics can fail in an oedematous kidney. This would presumably lead to worsening renal oedema and renal function, although the authors did not investigate this further (Katz, 1958).

#### Pyelolymphatic Backflow

A further explanation for the increased renal lymph flow seen in hydronephrosis is pyelolymphatic backflow. This has been shown experimentally in pigs and rabbits, and observationally in humans (Morrison, 1929; Narath, 1951; Cuttino et al., 1978; Bidgood et al., 1981). Retrograde ureteral injection of contrast under pressure was seen to rupture the fornices, fill the interstitium and drain via the cortical and cortico-medullary lymphatics (Cuttino et al., 1978). This has been portrayed as further evidence of the ‘safety valve’ mechanism of renal lymph (Goodwin and Kaufman, 1956). As opposed to lymphatico-venous communications, this is not a direct anatomical connection (unless there is a pathological fistula, e.g., parasitic chyluria) but rather a two-stage process where fluid is moved first into the interstitium and then into the lymphatics (Goodwin and Kaufman, 1956; Threefoot et al., 1987). The importance of lymphatic outflow in hydronephrosis can also be seen where metastatic obstruction of both the ureter and the renal lymphatic outflow results in ureteric rupture (Fergusson, 1943). O’Morchoe and O’Morchoe (1988) argued against pyelolymphatic backflow acting as a ‘safety valve’ in hydronephrosis, citing the fact that renal hilar lymph and obstructed pelvic urine have very different compositions (Holmes et al., 1977b), and the difference in composition becomes greater with prolonged obstruction, which is the opposite to what would be predicted if there was a ‘safety valve’ effect.

### Renal Interstitial Edema

**FIGURE 6:**
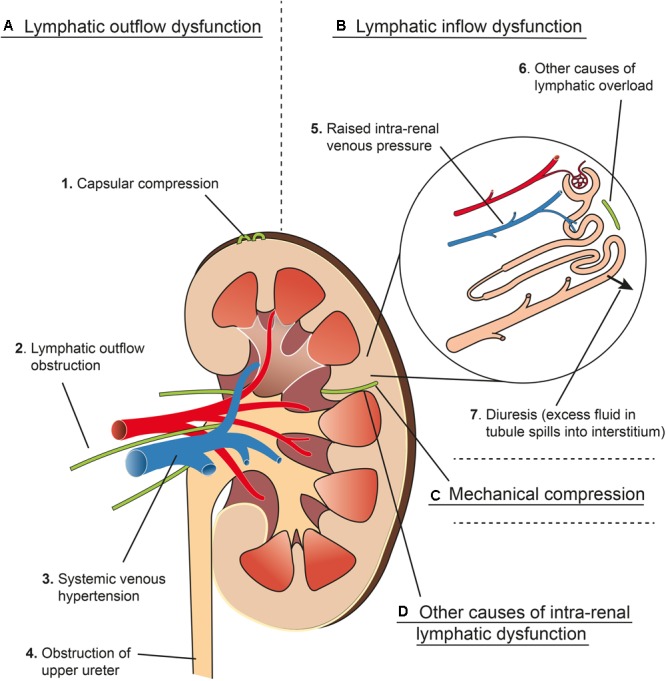
Schema representing four main mechanisms **(A–D)** that contribute to renal interstitial edema. **(A)** Lymphatic outflow dysfunction. (1) Capsular compression: Communicating and perforating lymphatics drain the superficial cortex under normal circumstances but can drain more of the kidney if the hilar route is obstructed. These lymphatics can become blocked by mechanical compression from a tense renal capsule from renal interstitial oedema (see Renal Lymph Flow During Hydronephrosis and Figure 4). (2) Lymphatic outflow obstruction: Lymphatics draining the kidney at the hilum can be obstructed experimentally or clinically by metastatic disease, or can be resected during renal transplant, which causes an effective obstruction of flow (see Renal Interstitial Edema and Post-renal Transplant). (3) Central venous hypertension: raised pressure at the subclavian vein can prevent effective drainage from the thoracic duct, leading to increased renal lymphatic outflow pressure (see Congestive Heart Failure). Central venous hypertension also increases intra-renal venous pressure (see **B5**). (4) Obstruction of the upper ureter causes distension of the renal pelvis, which compresses hilar veins and lymphatics, and may cause pyelolymphatic backflow (see Renal Lymph Flow During Hydronephrosis and Pyelolymphatic Backflow). **(B)** Lymphatic inflow dysfunction (lymphatic overload). (5) Raised intra-renal venous pressure causes increased interstitial fluid and lymphatic flow (see Renal Lymph Flow During Increased Venous Pressure). (6) Other causes of lymphatic overload: Initial lymphatic capillaries can be overwhelmed when there is excessive interstitial fluid for other reasons e.g., hypoproteinaemia (see Renal Interstitial Edema). (7) Diuresis causes increased lymphatic flow because excess fluid in the renal tubules results in more fluid reabsorbed into the interstitial space (see Renal Lymph Flow During Diuresis). **(C)** Mechanical compression of intra-renal collecting lymphatics: these may be compressed by renal interstitial edema because of the non-distensible nature of the renal capsule (see Relationship of Intra-renal Pressure to Renal Lymphatic Function). **(D)** Other causes of intra-renal lymphatic dysfunction such as incompetent valves (e.g., primary lymphatic disorders) or functional narrowing (e.g., lymphangio-spasm) (see Renal Interstitial Edema and Primary Renal Lymphatic Dysfunction). Arteries in red, veins in blue, lymphatics in green.

A summary of various factors that can lead to renal interstitial edema is outlined in Figure 6. Isolation and ligation of renal hilar lymphatics has been shown to cause renal interstitial edema, increased kidney size, increased tissue pressure, diuresis and naturesis (Kaiserling and Soostmeyer, 1939; Goodwin and Kaufman, 1956; Stolarczyk and Carone, 1975; Wilcox et al., 1984). Wilcox et al. (1984) showed that the observed changes were not due to accidental renal nerve ligation, changes in renal hemodynamics (e.g., GFR) or changes in osmolarity.

Goodwin and Kaufman (1956) argued that renal interstitial edema is a result of renal lymphatics reaching their capacity to carry away interstitial fluid. They list the factors that can lead to this as (i) mechanical obstruction of the lymphatics (e.g., tumor), (ii) functional lymphatic narrowing (e.g., lymphangio-spasm), (iii) excessive lymph flow (e.g., hypoproteinaemia), or (iv) pathological alterations in the lymphatic vessels themselves (e.g., primary lymphatic valvular disorders or transected lymphatic vessels post-transplant).

#### Relationship Between Renal Lymphatic Dysfunction and Renal Function

Renal lymphatic dysfunction, defined as a failure in adequate drainage, leading to renal interstitial edema, would be expected to decrease renal function due to pressure changes within the encapsulated kidney (Mullens et al., 2017). Indeed, increased pressure within the renal capsule leads to a decreased GFR and renal perfusion (Jaffee et al., 2018). Feder and McDonald (1967) measured renal function after obstructing all hilar and capsular lymphatic in dogs. As expected, the kidney became swollen and tense, and urinary output and urinary sodium both increased. Surprisingly, they also found a slightly increased creatinine clearance and p-aminohippurate clearance in the obstructed kidney. They speculated that this is due to the autoregulation of renal blood flow, where increased tissue pressure causes compression of the renal vascular bed and subsequent reflex hyperemia.

O’Morchoe and O’Morchoe (1988) suggested there was no specific evidence to indicate a special interdependent relationship between renal function and the renal lymphatic system. They cited the practical problem of occluding renal hilar lymphatics without damage to renal nerves. They also highlighted the fact that most previous studies have simply investigated the flow and composition of renal lymph under various conditions (i.e., diuresis, ureteric obstruction, raised renal venous pressure), and suggested all changes could simply be attributable to altered hemodynamic effects. They concluded that the lymphatic system functions in the kidney as it does elsewhere in the body, to remove accumulated protein and fluid from the interstitial spaces, but it remains unknown as to what effect removing the lymphatic system has on renal function.

Bell and Parry (1974) also do not consider that the renal lymphatic system has a specific impact on renal function. In canine kidneys, they ligated all observable hilar lymphatics as well as the fatty tissue at both poles of the kidney to obstruct the capsular lymphatics. In contrast to the experiments described earlier, neither subcapsular pressure nor IRVP increased after lymphatic ligation. Furthermore, they repeated the experiment after partial obstruction of the renal vein and noted no difference between lymphatic ligation and controls when IRVP was raised. They concluded that renal lymphatic vessels are not primarily involved with regulation of intra-renal pressures. They conceded that lymphatic ligation leads to interstitial edema but suggested this may be more pronounced in the rabbit (where there is no capsular lymphatic communication) than the dog. The histological changes seen with lymphatic ligation, they suggest, are due to interstitial accumulation of toxic products of metabolism rather than pressure related changes. The findings of Bell and Parry seem to contradict other studies (e.g., Wilcox et al., 1984) and it is possible they were not able to ligate all lymphatics. Feder and McDonald (1967) found many accessory lymphatic channels that were previously unrecognized opened up after ligating all observable hilar and capsular lymphatics in dogs.

#### Relationship of Intra-Renal Pressure to Renal Lymphatic Function

In studying the interplay between renal venous pressure, interstitial pressure and lymph flow, Rohn et al. (1996) devised a simple experimental model in dogs, where lymph flow (Q_L_) was measured at various outflow pressures (P_O_). From this they were able to calculate the single pressure source (P_L_) pushing renal lymph through a single resistance (R_L_). As expected they found a higher P_L_ when the renal venous pressure increased. Importantly they found a plateau in the Q_L_ versus P_O_ relationship, where there was no change in lymph flow as outflow pressure decreased from 5 to -5 cmH_2_O. This plateau extended when venous pressure was increased (up to 27.2 cmH_2_O), such that there was no change in lymph flow when outflow pressure decreased from 20 to -5 cmH_2_O. Because of this extension in the Q_L_ vs. P_L_ plateau, they reasoned that renal interstitial pressure may partially collapse *intra-renal collecting* lymphatics (not *initial capillary* lymphatics which are tethered via anchoring filaments to the interstitium), which in turn may compromise lymph flow. This has the important implication of suggesting that beyond a critical point of interstitial edema, lymph flow will not increase and edema, and renal function, will significantly worsen. Interestingly the same plateau was seen in the lymphatic flow of the lung in the dog (Drake et al., 1982) and sheep (Drake et al., 1985) but the effect was less marked. This may be a reflection of the less distensible nature of the renal capsule. The other important finding of their paper is that increased pressure at the outflow end of renal lymphatics (mimicking raised systemic venous pressure), significantly decreases renal lymph flow, which may have implications for patients with acute and critical illness who have a raised central venous pressure.

### Renal Lymphatic Function in Various Disease States

#### Congestive Heart Failure

Congestive heart failure (CHF) causes an increase in systemic venous pressure, which has multiple effects on renal lymph. Elevated venous pressures place a double burden on renal lymphatic vessels by increasing post-glomerular capillary filtration and opposing thoracic lymphatic drainage in the neck, which can cause backpressure effects, which decrease renal lymphatic outflow (Drake et al., 1998). As previously outlined, increased IRVP leads to increased renal lymphatic pressure and flow due to increased capillary filtration (Dumont et al., 1963). It is possible impaired lymphatic drainage in the neck occurs because central venous congestion causes a functional obstruction at the thoracic duct ostial valve, although this is unproven at present (Ratnayake et al., 2018). The excess demand on the lymphatics can lead to failure of adequate lymphatic drainage (lymphatic dysfunction). Cuttino et al. (1989), in studying renal lymphatic anatomy, found they had to use autopsy specimens from patients with CHF because the lymphatics had dilated and the valves become incompetent. This was the only way they were able to inject hilar lymphatics with contrast material in a retrograde manner and study the lymphatic anatomy using microradiographic techniques. Overall, this suggests renal lymphatic drainage may become compromised in CHF, through impaired outflow and valvular incompetence.

Renal lymphatics also contribute to sodium retention and worsening of generalized edema in CHF. Partial occlusion of the IVC (resembling CHF) increases renal lymph flow and Na^+^ content, and decreases urinary Na^+^ and flow in a reciprocal manner (Katz and Cockett, 1959). This mechanism is thought to contribute to sodium and fluid retention in CHF by recycling Na^+^ and fluid rather than excreting them in the urine. The increased renal lymphatic flow also washes out interstitial proteins, which decreases colloid osmotic pressure in the renal interstitium and further promotes sodium reabsorption (Mullens et al., 2017).

External drainage of lymph in the presence of an elevated central venous pressure has been shown to reduce edema formation, without reducing venous pressure in the gut (Drake et al., 1998). This suggests that external thoracic duct drainage may be of therapeutic benefit to the kidney (as well as other organs) in CHF (Dumont et al., 1963).

#### Acute Kidney Injury

The clinical entities of AKI and renal interstitial edema are closely and reciprocally linked (Prowle et al., 2010). Renal interstitial edema can result from many factors, but is especially associated with renal lymphatic dysfunction (Figure 6). As well as being a consequence of AKI (due to renal inflammation and increased capillary permeability), renal interstitial edema can in itself lead to AKI. As the kidney is encapsulated, excess interstitial fluid leads to a disproportionate rise in intra-renal pressure and a subsequent decrease in renal blood flow and GFR (Firth et al., 1988). Renal decapsulation has been shown to reduce the incidence of AKI in patients with hemorrhagic shock requiring massive resuscitation (Stone and Fulenwider, 1977). Renal interstitial edema also impairs oxygen and metabolite diffusion by increasing diffusion distances (Prowle et al., 2010). Furthermore, in the encapsulated kidney, it may compress lymphatic drainage, commencing a viscous cycle that leads to worsening edema and renal function (see Relationship of Intra-renal Pressure to Renal Lymphatic Function). This all highlights the key role that renal lymphatics play in prevention of both AKI and renal interstitial edema.

Many studies have shown that renal venous congestion is also an important factor in the development of AKI, and even possibly more important than arterial insufficiency (Li et al., 2011; Jaffee et al., 2018). Typically, venous congestion results in an ischemic-type kidney injury (Jaffee et al., 2018). Renal venous congestion increases interstitial fluid and renal lymphatic flow (see Renal Lymph Flow During Increased Venous Pressure). Therefore, renal lymphatic dysfunction, where lymphatic drainage fails to meet demand for whatever reason, can be expected to worsen AKI in the setting of venous congestion (e.g., heart failure, aggressive fluid resuscitation, intra-abdominal hypertension) (Prowle et al., 2010).

#### Chronic Renal Failure

As is the case for AKI, renal lymphatics have a complicated relationship to the prevention, pathogenesis and resolution of chronic kidney diseases (CKDs). Most, if not all, CKDs are characterized by tubulointerstitial inflammation and widening of the interstitial space through extracellular matrix deposition, leading in turn to fibrosis (Seeger et al., 2012). Renal lymphatics are thought to contribute to the development of fibrosis itself (Yazdani et al., 2014). CCL21 is constitutively expressed by renal LECs and can attract CCR7-positive fibrocytes. Blocking this pathway has been shown to reduce renal fibrosis (Sakai et al., 2006), suggesting a causative role of renal lymphatics in the development of CKDs.

Renal interstitial edema is seen in almost all CKDs, usually associated with tubulointerstitial damage and hypoproteinaemia secondary to proteinuria (Yazdani et al., 2014). Similar to AKIs, it can be considered a consequence of CKD, and one of the factors that promotes it. This is due to tissue architecture disruption, increased hydraulic conductivity and remodeling of the interstitial matrix (Yazdani et al., 2014). Therefore, lymphatic dysfunction can contribute to the development of CKDs by this mechanism. Consequently, lymphangiogenesis is activated in CKDs as an attempt at resolution (see Lymphangiogenesis). Further research is required to fully elucidate the roles of renal lymphatics in both AKI and CKD.

#### Post-renal Transplant

Following renal transplantation, where the lymphatics are inevitably divided, the kidney increases in size due to interstitial edema (Goodwin and Kaufman, 1956). Pedersen and Morris (1970) studied this phenomenon in sheep. They noticed lymph production increases in the post-transplant kidney, especially so in graft rejection, and the increase in kidney size could be avoided if renal lymph was drained artificially. Transplanted kidneys show active lymphangiogenesis in humans and rats, and LECs that actively organize inflammatory nodules through expression of the CCL21 chemokine (Yazdani et al., 2014). It is currently unknown whether lymphangiogenesis is detrimental or beneficial to the transplanted kidney and further research is needed in this area (Yazdani et al., 2014).

#### Lymphangiogenesis

Newly formed lymph vessels (lymphangiogenesis) normally function as protection, helping to clear tissue edema and inflammatory infiltrates. Renal lymphangiogenesis is associated with renal fibrosis, inflammation and transplant rejection (Kerjaschki et al., 2004; Seeger et al., 2012; Yazdani et al., 2014). In CKDs, such as diabetic nephropathy, the degree of lymphangiogenesis appears to correlate with fibrosis more than inflammation (Sakamoto et al., 2009). In a rat proteinuric model, lymphangiogenesis occurred prior to macrophage influx, collagen deposition and interstitial fibrosis, suggesting a possible causal role in fibrosis (Yazdani et al., 2014). Lymphangiogenesis is also seen in rat models of hydronephrosis and hypertension (Suzuki et al., 2012; Kneedler et al., 2017). In humans, it is seen in end-stage renal failure and renal cell carcinoma (in tissue surrounding the tumor) (Ishikawa et al., 2006). It is also a feature of hydronephrosis in humans around interlobar and arcuate vessels, but not around interlobular vessels (Ishikawa et al., 2006). It is not seen in specimens of old or new renal infarction or acute tubular necrosis (Ishikawa et al., 2006). Therapies aimed at inducing lymphangiogenesis (e.g., 9-*cis* retinoic acid) have improved tissue swelling in animal models of lymphedema, and may have a role in treating renal interstitial edema as well (Choi et al., 2012; Yazdani et al., 2014). However, these therapies may potentially also be detrimental, as lymph vessels transport antigen-presenting cells to lymph nodes and LECs can initiate immune responses in and of themselves (e.g., following transplant) (Yazdani et al., 2014).

#### Long-Term Effects of Renal Lymphatic Ligation

The long-term effects of ligating hilar lymph vessels in humans can be seen in renal transplantation, where the donor kidney’s lymphatic vessels are ligated (Ranghino et al., 2015). These effects are also seen following lymphatic ligation for the treatment of severe chyluria (Zhuo et al., 2013). It has been shown that the human kidney can survive long-term after lymphatic ligation, presumably due to new lymphatic connections. However, the true effects of this are not known. Zhang et al. (2007a,b) studied the effects of renal lymphatic ligation in rats over 8 weeks. They ligated both hilar and capsular lymphatic vessels and included animals where one kidney was ligated and the other removed to resemble a single transplanted kidney. They found severe proteinuria 1 week after ligation, and from 2 weeks, renal function began to deteriorate with elevated serum creatinine and reduced creatinine clearance. Histologically the kidneys showed tubular damage, tubulointerstitial fibrosis and expansion of the mesangium, possibly due to enhanced activation of the TGF-β1/Smad signaling pathway. These changes worsened over the 8-week study period. Renal lymphatic ligation has also been shown to cause enhanced renal cell apoptosis (Zhang et al., 2009; Cheng et al., 2013). Sclerosis of the renal parenchyma has also been seen in rats 3 months after ligation of the thoracic duct (Umbetov, 1989), although it is difficult to ascribe cause and effect in this situation. Renal interstitial fibrosis has been seen and thought to develop as a consequence of unabsorbed renal interstitial edema in a human retrospective long-term study (Wehrmann et al., 1989).

#### Primary Renal Lymphatic Dysfunction

Primary lymphatic disorders are varied and the majority of reported cases do not have an identified mechanism. In most disorders, defects in relation to lymphatic valves are the cause of lymphatic dysfunction (Ballard et al., 2018). These patients generally present with bilateral lower limb lymphedema (e.g., Nonne-Milroy lymphedema, lymphedema praecox). There is a paucity of literature in regards to the renal effects of these various syndromes, but some are known to cause renal insufficiency and structural defects over time (Northup et al., 2003).

## Conclusion

Renal lymphatics, despite their abundance, have received little or no significant attention in recent years. Initial lymphatic capillaries (intralobular lymphatics) lie in the cortex, in close proximity to renal tubules and glomeruli, and drain the excess interstitial fluid and macromolecules that accumulate in the interstitial space. They join interlobular lymphatics, which do not possess valves, and so from there, lymph may follow the arcuate and interlobar lymphatic vessels toward the hilum, or penetrate the capsule to join capsular lymphatics. This dual exit from the kidney may be beneficial in lymphatic obstruction.

Renal lymph is mainly composed of capillary filtrate and is therefore formed in excess when IRVP is raised, such as in cardiac failure or aggressive fluid resuscitation. The other main driver of renal lymph formation is interstitial volume (and pressure), which can increase with increased capillary permeability (e.g., renal inflammation or SIRS). Failure of renal lymphatics to adequately drain interstitial fluid (lymphatic dysfunction) leads to renal interstitial edema. Edema may be exacerbated if the renal capsule becomes so tense that it blocks penetrating and communicating lymphatics as they exit the kidney through the capsule. Similarly, the hilar lymphatic route may become functionally obstructed as intra-renal edema and subsequent pressure changes cause collapse of arcuate and interlobar lymphatics. Failure of lymphatic drainage will thus exacerbate renal interstitial edema within this encapsulated organ, and we propose this is likely to lead to raised intra-renal pressure and contribute to renal dysfunction. This relatively unexplored pathological axis now awaits further clinical investigation.

## Author Contributions

JW, AP, and MI conceptualized the manuscript. PR reviewed the literature and drafted the manuscript. JH, JW, MI, and AP reviewed the manuscript, critically edited, and intellectually contributed.

## Conflict of Interest Statement

The authors declare that the research was conducted in the absence of any commercial or financial relationships that could be construed as a potential conflict of interest.

## Abbreviations

AKI: acute kidney injury
IRVP: intra-renal venous pressure
LEC: lymphatic endothelial cell
LYVE-1: lymphatic vessel hyaluronan receptor 1
SIRS: systemic inflammatory response syndrome
